# Fecal Contamination in Point of Use (POU) Drinking Water and Its Associated Factors in Ethiopia: Systematic Review and Meta‐Analysis; “Implications for SDG 6 and WASH Interventions”

**DOI:** 10.1002/hsr2.71445

**Published:** 2025-11-23

**Authors:** Gashaw Melkie Bayeh, Abathun Temesgen, Almaw Genet Yeshiwas, Tilahun Degu Tsega, Sintayehu Simie Tsega, Asaye Alamneh Gebeyehu, Getaneh Atikilt Yemata, Rahel Mulatie Anteneh, Getasew Yirdaw, Chalachew Yenew, Amare Genetu Ejigu, Ahmed Fentaw Ahmed, Zeamanuel Anteneh Yigzaw, Abebaw Molla Kebede, Habitamu Mekonen, Berhanu Abebaw Mekonnen, Meron Asmamaw Alemayehu, Aschale Shimels Alemu, Anley Shiferaw Enawgaw

**Affiliations:** ^1^ Department of Environmental Health, College of Medicine and Health Sciences Injibara University Injibara Ethiopia; ^2^ Department of Public Health, College of Medicine and Health Sciences Injibara University Injibara Ethiopia; ^3^ Department of Medical Nursing, School of Nursing, College of Medicine and Health Sciences University of Gondar Gondar Ethiopia; ^4^ Depatment of Public Health, College of Health Science Debre Tabor University Debre Tabor Ethiopia; ^5^ Department of Environmental Health Science, College of Medicine and Health Sciences Debre Markos University Debre Markos Ethiopia; ^6^ Department of Public Health Debre Tabor University Debra Tabor Ethiopia; ^7^ College of Veterinary Medicine and Life Sciences City University Hong Kong China; ^8^ Department of Midwifery, College of Medicine and Health Sciences Injibara University Injibara Ethiopia; ^9^ Department of Health Promotion and Behavioral Sciences, School of Public Health, College of Medicine and Health Sciences Bahir Dar University Bahir Dar Ethiopia; ^10^ Department of Human Nutrition, Collage of Health Sciences Debre Markos University Debre Markos Ethiopia; ^11^ Department of Nutrition and Dietetics, School of Public Health, College of Medicine and Health Sciences Bahir Dar University Bahir Dar Ethiopia; ^12^ Department of Epidemiology and Biostatistics, Institute of Public Health, College of Medicine and Health Sciences University of Gondar Gondar Ethiopia; ^13^ Department of Public Health, College of Health Sciences Debre Markos University Debre Markos Ethiopia

**Keywords:** drinking water, Ethiopia, fecal contaminations, Point of Use

## Abstract

**Background and Aims:**

Access to safe drinking water is a fundamental human right and a critical component of public health, particularly in developing countries like Ethiopia. This systematic review and meta‐analysis aimed to assess the prevalence of fecal contamination in Point of Use (POU) drinking water and identify its associated factors within the Ethiopian context, with implications for achieving sustainable development goal (SDG) 6 and enhancing water, sanitation, and hygiene (WASH) interventions.

**Methods:**

A comprehensive search across multiple databases yielded 12 studies, encompassing 5285 drinking water samples. Funnel plot and *I*² test assessed publication bias and heterogeneity. The DerSimonian and Laird random‐effects model estimated the pooled prevalence of fecal contamination in POU drinking water. Eggers and Beggs tests evaluated the small study effect, while subgroup and sensitivity analyses identified sources of heterogeneity.

**Results:**

The overall pooled prevalence of fecal contamination in POU drinking water was found to be 65.02% (95% CI: 56.33, 73.72), with significant heterogeneity (*I*² = 98.13%, *p* < 0.001). Fecal contamination in POU drinking water was significantly associated with environmental factors such as unimproved sanitation facilities [3.07, 95% CI: 2.59, 3.54] and unimproved water sources [3.03, 95% CI: 1.73, 4.32]; behavioral factors such as absence of household (HH) water treatment practices [4.08, 95% CI: 2.71, 5.45], unsafe withdrawal methods from storage container [3.15, 95% CI: 2.37, 3.93], and prolonged storage of drinking water [3.27, 95% CI: 1.13, 5.41].

**Conclusions:**

The findings underscore the urgent need for targeted interventions to improve water quality at the HH level, including education on safe water handling, effective treatment methods, and infrastructure improvements. These efforts are essential to mitigate the health risks associated with contaminated drinking water and to progress towards the SDGs in Ethiopia.

**Abbreviations**
AAU: Addis Ababa University
AJOL: African Journal Online
AOR: adjusted odds ratios
CI: confidence interval
DALYs: disability‐adjusted life years
EDHS: Ethiopian Demographic and Health Surveys
*E. coli*
: *Escherichia Coli*
GBD: global burden of diseases
GTP: growth and transformation plans
HHs: households
JBI: Joanna Briggs Institute
LMIC: low and middle income country
OR: odds ratios
POU: Point of Use
PROSPERO: International Perspectives Registrations of Systematic Reviews
SDGs: sustainable development goals
SDI: Socio‐Demographic Index
SSA: Sub‐Saharan Africa
UOG: University of Gondar
WASH: water sanitation and hygiene
WHO: World Health Organizations
WSPs: water safety plans

## 1 Introduction

Water is the most abundant resource on earth and it should be available to everyone, ensuring that no one is left behind [1]. Access to safe drinking water is acknowledged as a basic human right [2, 3], and the sustainable development goals (SDGs) emphasize the need for universal and equitable access to safe drinking water for every populations in member countries, including Ethiopia [4]. Ethiopia has successfully incorporated the SDGs into its Second Growth and Transformation Plan (GTP II), which was implemented from 2015/16 to 2019/20. Furthermore, the Ethiopian government has developed a 10‐year development plan for 2019/20 to 2029/30, ensuring complete alignment with the 2030 Agenda and the SDGs [4].

Despite the situation, millions in Ethiopia still rely on water sources that are untreated or poorly managed, creating major health risks [5]. About 32.5% of the Ethiopian population uses unimproved water sources, and 13.5% get their drinking water from surface water [6]. This highlights an urgent need for water treatment within HHs. Sadly, only less than 10% of Ethiopian HHs utilize effective water treatment methods [7, 8]. These circumstances contribute to a substantial burden of diarrheal diseases, which have serious implications for public health.

According to the global burden of disease (GBD), between 1990 and 2019, the number of deaths by diarrheal diseases related to unsafe water was decreased by 50%, while the rate of disability‐adjusted life years (DALYs) was declined by 59% [9]. Despite this progress, diarrheal diseases were still very common in areas with low Socio‐Demographic Index (SDI) levels, particularly in Africa. This is mainly due to interconnected problems, with about 69% of diarrhea cases attributed to poor water, sanitation, and hygiene (WASH) practices [10]. Diarrheal diseases are a significant concern for public health in Ethiopia, greatly impacting the rates of illness and death, particularly among children under 5 years old. They are the second leading cause of DALYs, with a rate of 2592.5 per 100,000, following lower respiratory infections [11].

The quality of drinking water is a crucial element in the spread of diarrheal diseases linked to WASH problems, particularly in developing nations like Ethiopia [12, 13]. The contamination of drinking water throughout its journey from the source to the Point of Use (POU) in HHs is responsible for the deterioration of its microbial quality, posing serious challenges to the realization of the SDGs in the country [14]. The microbial quality of drinking water at the POU is influenced by various factors, including environmental, behavioral, infrastructural, and socioeconomic conditions [15]. Drinking water source contamination can occur from runoff during heavy rains [16], and when water sources are near livestock or septic systems [17]. Poor sanitation in rural areas increases risks of contaminations [18]. Additionally, how people collect, store, and treat water can either reduce or increase contamination [19, 20, 21]. Practices like using unclean storage containers, leaving water uncovered and withdrawing water by dipping with unclean cans can lead to microbial contamination at home [14, 15, 22].

Despite improvements in access to water and sanitation, drinking water in many Ethiopian HHs remains vulnerable to contamination, resulting in higher rates of waterborne diseases. The water that has been treated both at the source and at the HH level often becomes re‐contaminated due to poor storage and handling practices. Therefore, monitoring water quality at the point of consumption is essential for accurately evaluating the immediate public health risks associated with drinking contaminated water [23]. This complements source water monitoring by providing a more complete picture of potential exposure and ensuring safety where it matters most. This is a critical step in achieving SDG 6 by designing appropriate interventions to ensure that water is safe for drinking, considering the potential for contamination during transport and storage.

Several studies have examined HH water treatment methods, handling practices, and drinking water quality within the Ethiopian context. Some of these studies highlighted that microbial contamination at the point of consumption is a significant problem in Ethiopia. However, ineffective and fragmented interventions have been implemented due to a lack of summarized data that could inform evidence‐based targeted interventions. Therefore, considering the importance of synthesis of existing evidence, this systematic review and meta‐analysis aimed to identify and analyze the key risk factors affecting the microbial quality of drinking water at the POU in Ethiopia, which may have an input in ensuring SDGs in the country.

## 2 Methods

### 2.1 Review Question

We have used **CoCoPop** as mnemonics review questions, where, The **Condition (Co):** Point of drinking water microbial quality; The **Context (Co**)**:** in Ethiopia; The **Population (Pop)**: Household level.

### 2.2 Protocol Registration

This study has been promptly registered with the International Prospective Register of Systematic Reviews (PROSPERO) to enhance transparency and reduce the risk of bias. The PRISMA checklist guideline was used to ensure study's quality and result validity, enhancing its credibility [24].

### 2.3 Search Strategy and Database

A comprehensive search strategy was used across multiple databases, including PubMed, Web of Science, Scopus, Cochrane Library, Google Scholar, and African Journals Online (AJOL). The search strategy used keywords and phrases connected with Boolean operators as follows: (((((((((((Drinking water) OR (Water)) AND (Microbial quality)) OR (Bacteriological quality)) OR (Fecal contamination)) OR (*E. coli* contamination)) AND (Associated factors)) OR (Risk Factors)) OR (Determinant Factors)) AND (Point of use)) AND (Ethiopia)). Inclusion and exclusion criteria were set to maintain the quality of included studies. Additionally, a manual search of reference lists and online repositories from the University of Gondar (UoG) and Addis Ababa University was done to find more literatures (Supporting Information S1: File S1).

### 2.4 Study Selection

The search strategy of the review was managed and arranged by EndNote version 8. Three authors (GM, AG, and AT) conducted an independent review of the included studies, during which duplicates of the articles were identified and removed. The titles and abstracts of the remaining articles were then reviewed to see if they satisfied the established inclusion and exclusion criteria. The full texts of the articles were also examined to determine the best and most pertinent literatures that were eventually included in this review. Disagreements among the reviewing authors during the article selection process were discussed and resolved by considering the relevance of the studies, their alignment with the goals of this review, and their adherence to the inclusion requirements. Additionally, if the disputes are not resolved through conversation based on objective standards, expert advice was sought. Finally, the three authors agreed upon the articles that were included in this review. The processes of article selection tried to include a rigorous, unbiased selection process and ensured the inclusion of high‐quality studies in the review.

### 2.5 Eligibility Criteria (Inclusion and Exclusion Criteria)

#### 2.5.1 Inclusion Criteria

This review included full‐text articles that were published in English to avoid problems of inconsistencies during interpretation of results. Full‐text articles provided sufficient data, such as proportions, incidence rates, and odds ratios (ORs) related to the microbial quality of water at the POU. Time period restrictions was not made so that all relevant articles were included.

#### 2.5.2 Exclusion Criteria

This review excluded articles that were not in full‐text format after the corresponding authors repeatedly declined our requests for full‐text access. Studies with insufficient data for key results such as the number of participants, and high risk of bias, or unclear methods for outcome assessment were also excluded.

### 2.6 Operational Definitions

#### 2.6.1 POU Drinking Water

Water collected from various sources and made available for immediate drinking in HHs.

#### 2.6.2 Fecal Contamination in POU Drinking Water

The presence of specific microbial indicators, i.e., either *E. coli* or thermo‐tolerant bacteria in a water that is made available for immediate drinking in HHs [25].

### 2.7 Data Extraction

Rigorous data extraction and reconciliation methods was used to ensure its accuracy, consistency, and reliability. A structured data extraction format was developed using Microsoft Excel 2016. Two authors (GM and AG) independently utilized this tool to record details including the primary authors’ names, publication year, study year, season of the study, sampling method, study setting, sample size, response rate, prevalence of fecal coliform indicator bacteria at the drinking point, and the ORs with their confidence intervals (CIs) for relevant factors. Disagreements in the results of data extraction process were handled by discussions between the two authors, with the third author (AT) verifying if needed. The prevalence of fecal coliform indicator bacteria at point of drinking was extracted or calculated by dividing the number of samples tested positive for fecal coliforms with the total number of samples tested from the included studies. The adjusted odds ratios (AOR) were directly extracted from the included studies (Supporting Information S2: File S2).

### 2.8 Quality Assessment (Criticism and Evaluation of Quality)

The Joanna Briggs Institute (JBI) quality appraisal checklist designed for cross‐sectional and prevalence studies was used to evaluate the methodological quality of included studies. Two authors (GM & AG) independently evaluated all studies for their quality, research comparability, and strength of statistical analysis. The authors discussed disagreements on quality evaluation results and reached a consensus on study inclusion. A quality evaluation score of 50% or higher was needed for a study to be included in this review, as it indicated an acceptable lower risk of bias (Tables 1 and 2).

**Table 1:** Assessment of the methodological quality of included studies using the JBI critical appraisal checklist for analytical cross‐sectional studies.

No.	Author year	JBI quality assessment checklist for analytical studies								
		Q1 = Were the criteria for inclusion in the sample clearly defined?	Q2 = Were the study subjects and the setting described in detail?	Q3 = Was the exposure measured in a valid and reliable way?	Q4 = Were objective, standard criteria used for measurement of the condition?	Q5 = Were confounding factors identified?	Q6 = Were strategies to deal with confounding factors stated?	Q7 = Were the outcomes measured in a valid and reliable way?	Q8 = Was appropriate statistical analysis used?	Quality score (%)
1.	Alemeshet et al. 2021 [26]	Yes	Yes	Yes	Yes	Yes	Yes	Yes	Yes	100
2.	Getachew et al. 2021 [27]	Yes	Yes	Yes	Yes	Yes	Yes	Yes	Yes	100
3.	Fentie et al. 2024 [15]	Yes	Yes	Yes	Yes	Yes	Yes	Yes	Yes	100
4.	Mekonnen et al. 2019 [28]	Yes	Yes	Yes	Yes	Yes	Yes	Yes	Yes	100
5.	Berihun et al. 2023 [29]	Yes	Yes	Yes	Yes	Yes	Yes	Yes	Yes	100
6.	Getachew et al. 2018 [30]	Yes	Yes	Yes	Yes	Yes	Yes	Yes	Yes	100
7.	Birhan et al. 2022 [31]	Yes	Yes	Yes	Yes	Yes	Yes	Yes	Yes	100
8.	Aydamo et al. 2024 [32]	Yes	Yes	Yes	Yes	Yes	Yes	Yes	Yes	100

**Table 2:** Assessment of the methodological quality of included studies using the JBI critical appraisal checklist for prevalence studies.

No.	Author year	JBI quality assessment checklist for prevalence studies									
		Q1 = Was the sample frame appropriate to address the target population?	Q2 = Were study participants sampled in an appropriate way?	Q3 = Was the sample size adequate?	Q4 = Were the study subjects and the setting described in detail?	Q5 = Was the data analysis conducted with sufficient coverage of the identified sample?	Q6 = Were valid methods used for the identification of the condition?	Q7 = Was the condition measured in a standard, reliable way for all participants?	Q8 = Was there appropriate statistical analysis?	Q9 = Was the response rate adequate, and if not, was the low response rate managed appropriately?	Quality score
1.	Keleb et al. 2022 [33]	Yes	Yes	No	Yes	No	Yes	Yes	Yes	Yes	77.78
2.	Usman et al. 2018 [14]	Yes	Yes	Yes	Yes	Yes	Yes	Yes	Yes	Yes	100
3.	Feleke et al. 2018 [34]	Yes	Yes	No	Yes	Yes	Yes	Yes	Yes	Yes	88.89
4.	Amenu et al. 2014 [35]	Yes	Yes	No	Yes	Yes	Yes	Yes	Yes	Yes	88.89

### 2.9 Statistical Analysis and Data Aggregation

Data extracted with Microsoft Excel spreadsheet was exported to STATA version 17 software for further statistical analysis. A random‐effects model (DerSimonian and Laird) was employed under the assumption that the included studies do not estimate the same underlying effect size. This approach accounts for variability by recognizing that the true effect sizes differ across studies, which allows for a more accurate representation of the data. However, the inclusion of studies with varying effect sizes may inadvertently introduce publication bias, where studies with significant findings are more likely to be published. The Cochrane Q statistic and the *I*² index were used to evaluate the heterogeneity between studies. A value of *I*² ≤ 25% and *I*² ≥ 75% considered to indicate low and high heterogeneity, respectively. A *p*‐value less than 0.05 in Cochran's *Q* test was considered as there is significant heterogeneity.

### 2.10 Handling Publication Bias

To evaluate potential publication bias, Begg's rank correlation test and Egger's intercept test were employed, with a *p*‐value less than 0.05 indicating possible bias. A funnel plot was visually inspected to assess the symmetry of the included studies. When asymmetry was observed and Egger's test yielded a *p*‐value below 0.05, a trim‐and‐fill analysis was conducted to estimate the number of missing studies and adjust the overall effect size accordingly.

### 2.11 Subgroup and Sensitivity Analysis

The subgroup analysis was performed to determine the source of heterogeneity (*I*² index ≥ 50%) in data. Factors considered included the season of data collection (dry vs. wet), study year, study setting (urban vs. rural), water source type (improved vs. unimproved), study region and type of tests used (*E. coli* vs. fecal coliform) to better understand the data and improve water quality at the point of drinking. The sensitivity analysis was also conducted to see the effect of single study on the overall pooled prevalence estimates and increase robustness of the findings.

### 2.12 Displaying Results

The results of this meta‐analysis were displayed using a forest plot, which provided a clear visual presentation of the pooled effect sizes and their 95% CIs. The pooled AOR was used as the measure of effect size to identify variables predicting fecal contamination in POU drinking water in Ethiopia.

### 2.13 Ethical Approval and Consent to Participate

Ethical approval and consent to participate were not applicable for this study.

## 3 Results

### 3.1 Study Selection and Identification

**Figure 1:**
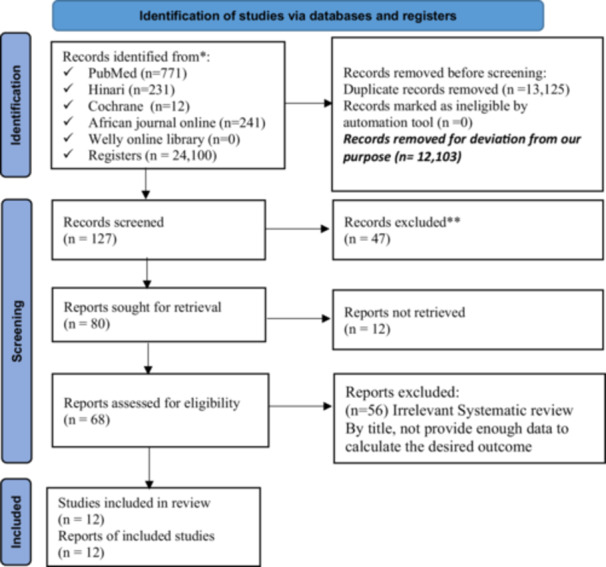
PRISMA flow diagram for selection of included studies for systematic review and meta‐analysis on fecal contamination in POU drinking water and its associated factors in Ethiopia.

A total of 25,355 studies or records were found from different electronic online databases. Of these, 13,125 studies were excluded due to duplication, and 12,103 records were removed due to deviation from the objectives of this review. Additionally, 85 studies were excluded after reviewing their titles and abstracts because of they were irrelevant for this review, and 115 studies were removed due to poor quality and a lack of clearly defined outcomes. Finally, 12 studies were included to assess the pooled effect size of fecal contamination in drinking water and its associated factors in Ethiopia (Figure 1).

### 3.2 Characteristics of Included Studies

This systematic review and meta‐analysis incorporated a total of 5285 drinking water samples drawn from 12 selected studies. Majority 8 (66.67%) of included studies were from Amhara region [14, 15, 27, 29, 30, 31, 33, 34], while the rest four studies were from Harari [26], Gambella [28], Oromia [35], and SNNP [32] regions.

### 3.3 Prevalence of Fecal Contamination in POU Drinking Water in Ethiopia

**Figure 2:**
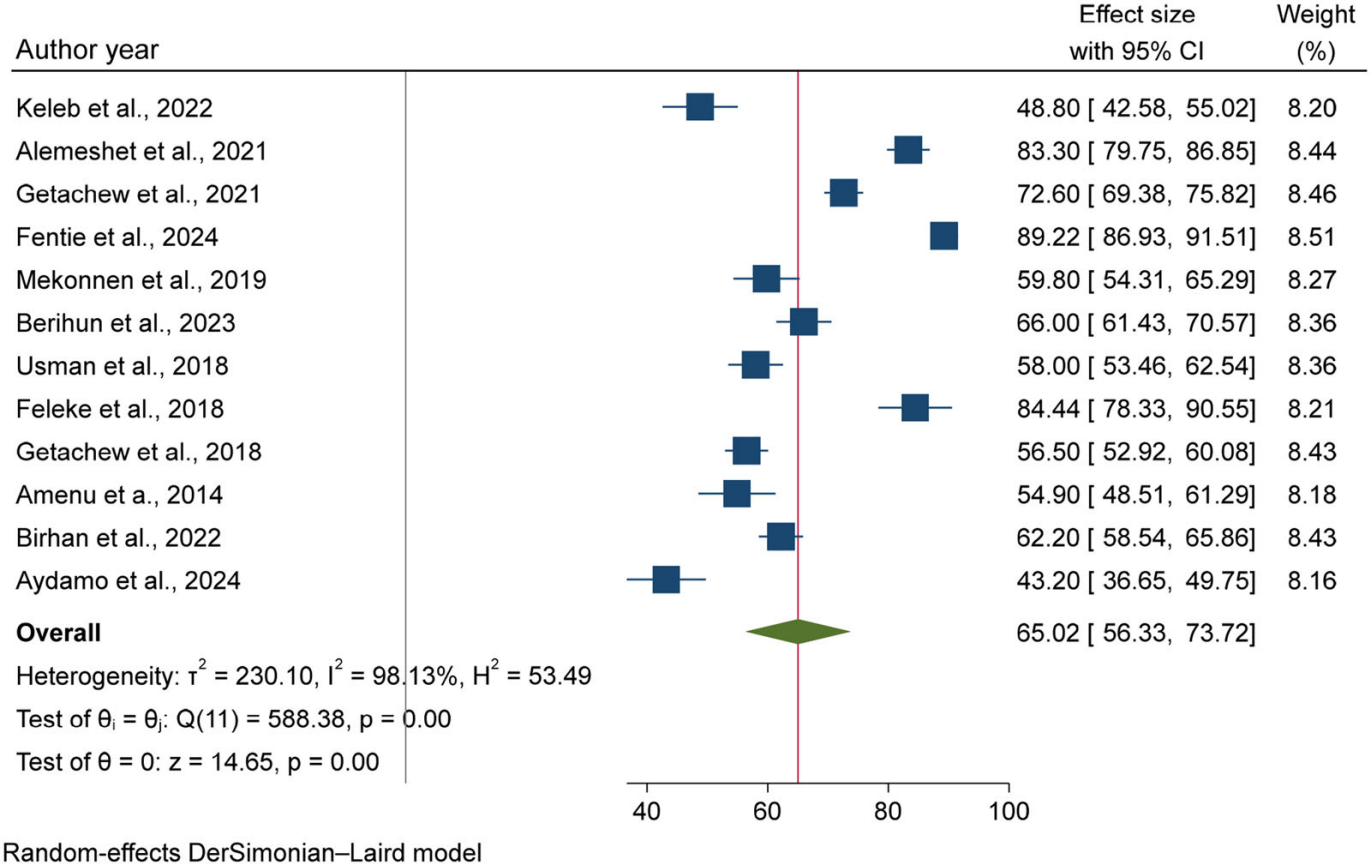
The forest plot of the pooled prevalence of fecal contamination in POU drinking water in Ethiopia, 2024.

The overall pooled prevalence of fecal contaminations in POU drinking water in Ethiopia was 65.02% [95% CI: 56.33, 73.72], with between studies heterogeneity (*I* ^2%^ = 98.13%, *p* < 0.001) (Figure 2).

#### 3.3.1 Heterogeneity and Publication Bias

The Cochrane heterogeneity value (*I* ^2%^ = 98.13%, *p* < 0.001) revealed substantial variability among the included studies. To explore possible sources of this heterogeneity, subgroup analysis were performed. Additionally, sensitivity analysis were conducted to assess the impact of individual studies on the overall pooled effect size and to enhance the robustness of the findings.

#### 3.3.2 Sub‐Group Analysis

The microbial quality of drinking water at the POU is influenced by various factors, including seasonal variation, water source type, geographic regions, and differences between urban and rural settings. To assess their impact, we performed subgroup analysis based on the data collection season (dry vs. wet), study setting (urban vs. rural), water source type (improved vs. unimproved), region of the study, and type of tests used (*E. coli* vs. fecal coliform).

**Figure 3:**
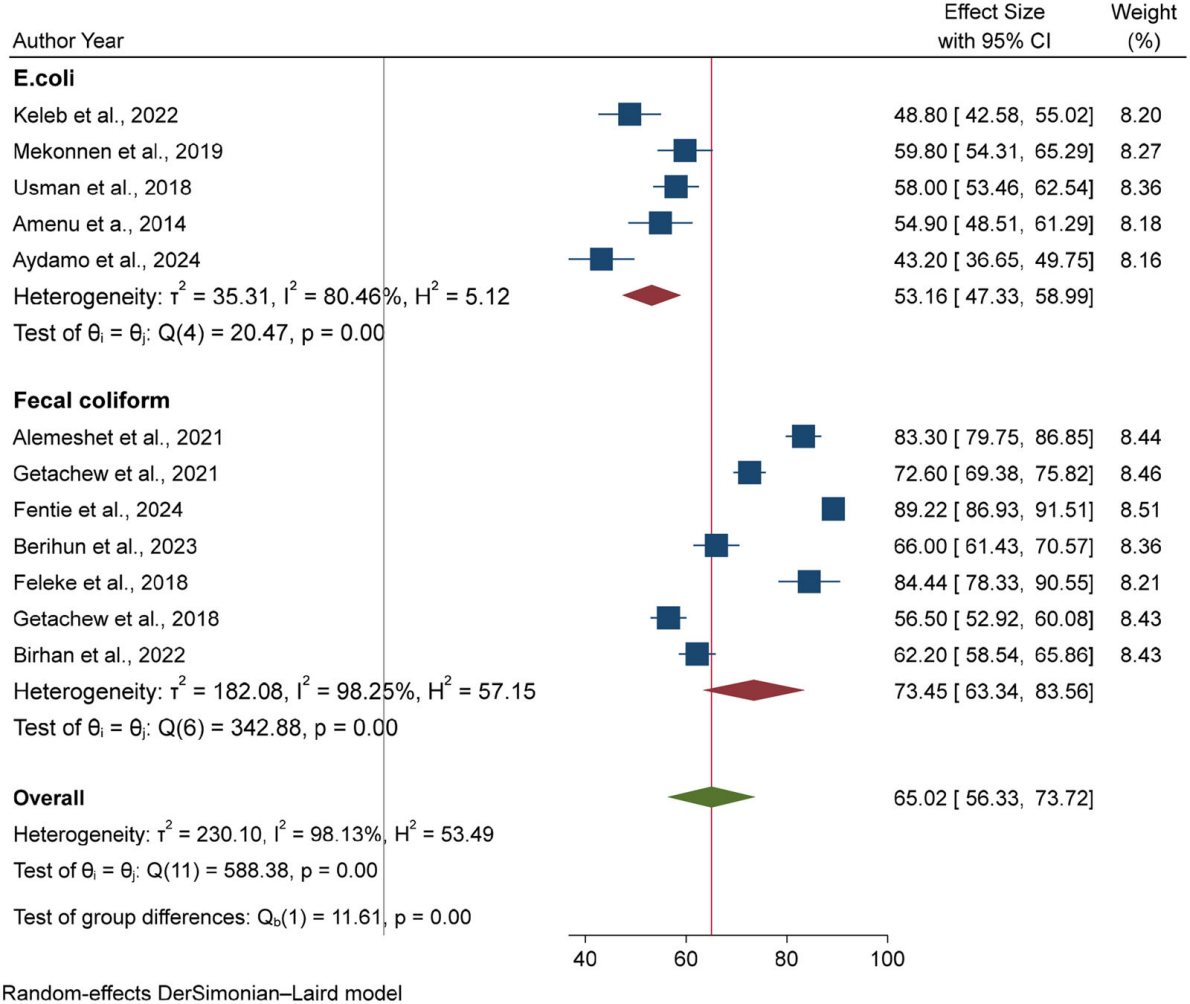
Sub‐group analysis to explore the source of heterogeneity stratified by the type of water sample tests used.

Our analysis identified the type of microbial test used as the primary source of heterogeneity. Studies measuring fecal coliform reported higher contamination rate of 73.45% [95% CI: 63.34, 83.56], compared to those measuring *E. coli*, which showed contamination rate of 53.16% [95% CI: 47.33, 58.99] (Figure 3).

#### 3.3.3 Sensitivity Analysis

**Figure 4:**
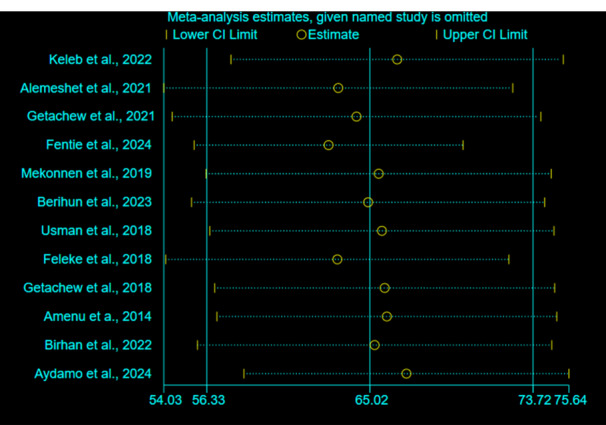
Sensitivity analysis of the pooled prevalence of fecal contamination in POU drinking water in Ethiopia.

Sensitivity analysis using the random‐effects model revealed that no individual study significantly influenced the overall effect size (Figure 4).

#### 3.3.4 Publication Bias

**Figure 5:**
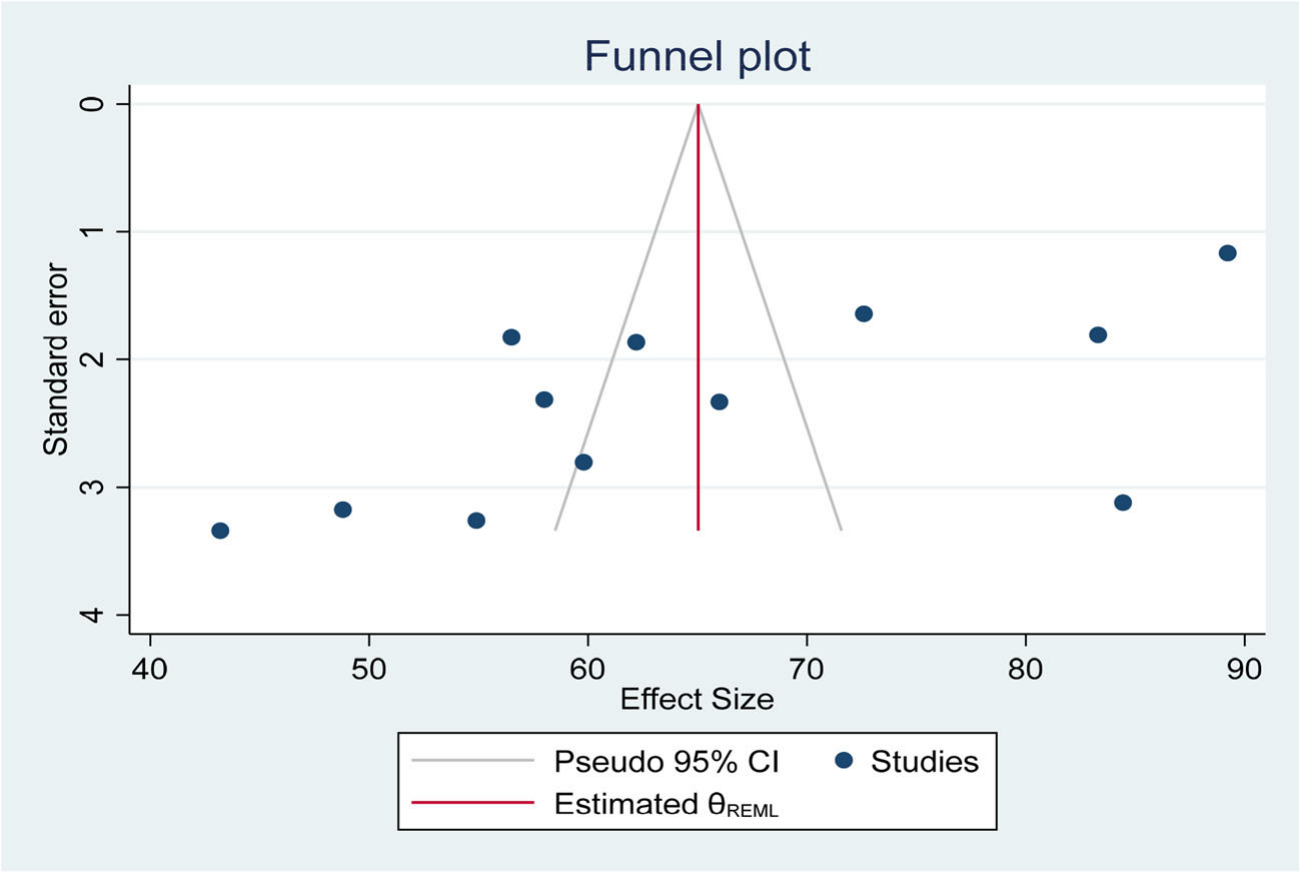
Funnel plot to show the presence of publication bias on the pooled prevalence of fecal contamination in POU drinking water in Ethiopia.

The evidence of publication bias, attributed to small‐study effects, was identified through asymmetry in the funnel plot. Therefore, a trim‐and‐fill analysis was conducted to adjust for potential bias and visualize the funnel plot with imputed studies (Figure 5).

Furthermore, the Egger's test showed that there is evidence of small study effect (*B* = −15.33897, *p* = 0.010) (Table 3). However, the Begg's test (Kendall's score = −24.00, *p* = 0.1148) showed that there is no small study effect.

**Table 3:** The eggers test to diagnose the effect of small study on the pooled prevalence of fecal contamination in POU drinking water in Ethiopia.

Std. Eff	Coefficient	Std. err	*T*	*p* > |*t*|	[95% Confidence interval]	
Slope	101.0576	9.804731	10.31	0.000	79.21134	122.9039
Bias	−15.33897	4.801726	−3.19	0.010	−26.03789	−4.640062

*Note:* Test of H0: no small‐study effects *p* = 0.010.

#### 3.3.5 Meta Regression (Source of Publication Bias)

Meta regression was computed to identify sources of publication bias as both Egress test and funnel plot analysis revealed that an evidence of publication bias. The analysis revealed that the observed bias was primarily attributable to differences in microbial contamination measurement methods, i.e., fecal coliform versus *E. coli*. A statistically significant higher prevalence of fecal contamination of drinking water at the POU was found in studies measuring fecal coliform compared to those measuring *E. coli* (*p* = 0.009) (Table 4).

**Table 4:** The meta regression to detect the source of publication bias on the pooled prevalence of fecal contamination in POU drinking water in Ethiopia.

Meta estimate variables	Coefficient	Std. errs.	*Z*	*p* > |*z*|	[95% Confidence interval]	
					LCI	UCI
Season of sampling						
Dry	Ref	Ref	Ref	Ref	Ref	Ref
Wet	12.1859	12.99075	0.94	0.348	−13.27549	37.6473
Dry & Wet	8.985191	7.113522	1.26	0.207	−4.957055	22.92744
Water source type						
Improved	Ref	Ref	Ref	Ref	Ref	Ref
Improved & Unimproved	−2.966077	7.199991	−0.41	0.680	−17.0778	11.14565
Test type						
*E. coli*	Ref	Ref	Ref	Ref	Ref	Ref
Fecal coliform	19.87291	7.561199	2.63	0.009	5.053234	34.69259
Constant	50.58018	7.042315	7.18	0.000	36.77749	64.38286

### 3.4 Factors Associated With Fecal Contaminations in POU Drinking Water in Ethiopia

**Figure 6:**
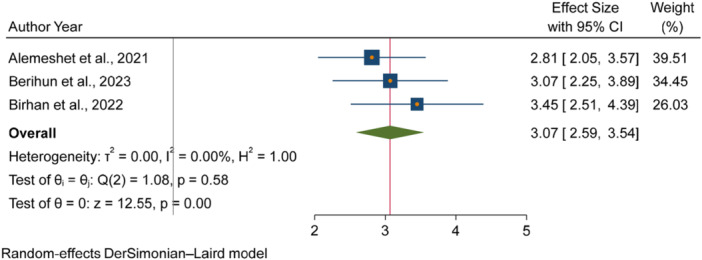
Forest plot showing the association between use of unimproved sanitation facilities and fecal contamination in POU drinking water in Ethiopia.

**Figure 7:**
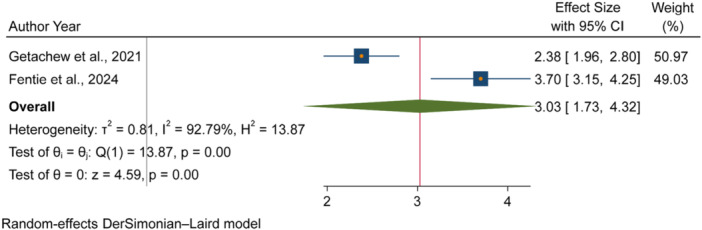
Forest plot showing the association between water collected from unimproved water sources and fecal contamination in POU drinking water in Ethiopia.

**Figure 8:**
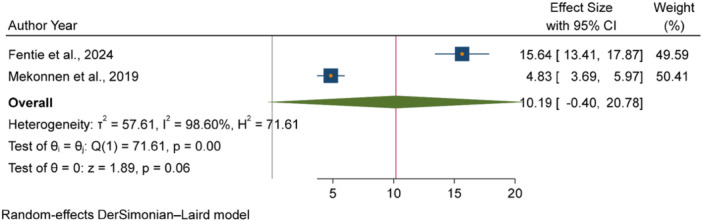
Forest plot showing the association between use of surface water as water source and fecal contamination in POU drinking water in Ethiopia.

**Figure 9:**
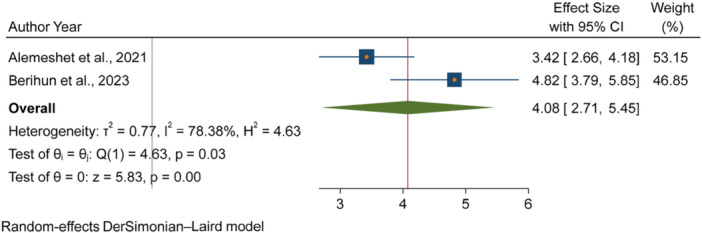
Forest plot showing the association between inadequate HH water treatment and fecal contamination in POU drinking water in Ethiopia.

**Figure 10:**
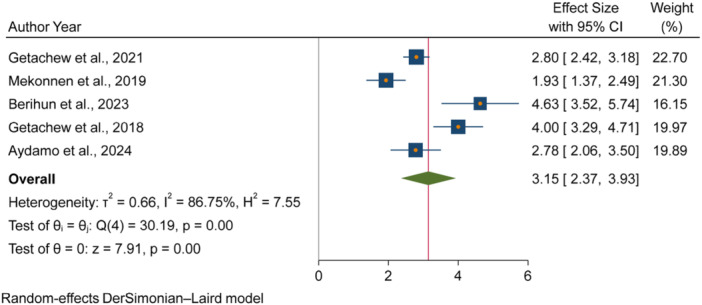
Forest plot showing the association between HH water storage practices and fecal contamination in POU drinking water in Ethiopia.

**Figure 11:**
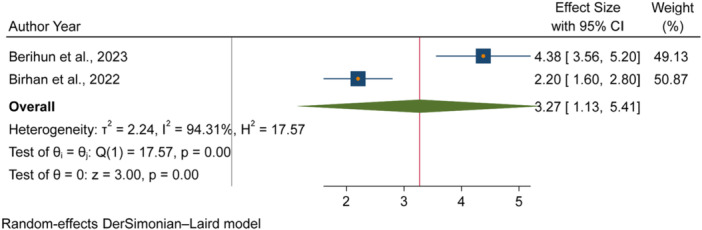
Forest plot showing the association between unsafe method of withdrawing water from storage container and fecal contamination in POU drinking water in Ethiopia.

The pooled odds of multiple studies demonstrated significant associations between increased fecal contamination in POU drinking water and both environmental and behavioral related factors. Environmental related factors included the use of unimproved sanitation facilities [26, 29, 31] (Figure 6), and water collected from unimproved water sources [15, 27] (Figure 7). In contrast, the use of surface water as a drinking source [15, 28] did not show statistically significant association with fecal contamination in POU drinking water (Figure 8). Behavioral factors contributing to fecal contamination in POU drinking water included the absence of HH water treatment practices [26, 29] (Figure 9), prolonged water storage habits [27, 28, 29, 30, 32] (Figure 10); and unsafe method of withdrawing water from storage container [29, 31] (Figure 11).

Accordingly, HHs with unimproved sanitation facilities [26, 29, 31] had 3.07 times [3.07, 95% CI: 2.59, 3.54] higher fecal contamination in POU drinking water compared to those with improved sanitation facilities (Figure 6). Similarly, HHs collecting water from unimproved sources [15, 27] had 3.03 times [3.03, 95% CI: 1.73, 4.32] higher fecal contamination in POU drinking water compared to those who collect from improve sources (Figure 7). Additionally, HHs lacking water treatment practice [26, 29] had 4.08 times [4.08, 95% CI: 2.71, 5.45] higher fecal contamination in POU drinking water than HHs that treated their drinking water (Figure 9). Those HHs with prolonged water storage habits [27, 28, 29, 30, 32] had 3.15 times [3.15, 95% CI: 2.37, 3.93] higher fecal contamination in POU drinking water compared to those HHs without prolonged water storage habits (Figure 10). Those HHs employing unsafe methods for withdrawing water from storage containers [29, 31] had 3.27 times [3.27, 95% CI: 1.13, 5.41] higher fecal contamination in POU drinking water compared to those using safe withdrawal practices (Figure 11).

However, use of surface water as water source [15, 28] did not show significant association with fecal contamination of water at point of drinking (Figure 8).

## 4 Discussion

According to the World Health Organization (WHO), in 2022, at least 1.7 billion people globally relied on drinking water source contaminated with fecal matter [36]. The pooled prevalence of fecal contamination in POU drinking water in Ethiopia was 65.02% [95% CI: 56.33, 73.72], with among studies heterogeneity (*I* ^2%^ = 98.13%, *p* < 0.001). The substantial heterogeneity observed across studies (*I*² = 98.13%, *p* < 0.001) was attributed to differences in the microbial testing methods employed (*E. coli* vs. Fecal coliform). Subgroup analysis showed that studies measuring fecal coliform reported higher fecal contamination in POU drinking water (73.45%; 95% CI: 63.34, 83.56) compared to those measuring *E. coli* (53.16%; 95% CI: 47.33, 58.99). Similarly, meta‐regression analysis confirmed that studies measuring fecal coliform had higher prevalence of fecal contamination in POU drinking water compared to those measuring *E. coli* (*p* = 0.009). This might be due to the broader range of bacterial species included in fecal coliforms than *E. coli* alone. Fecal coliforms represent a group of bacteria that include several species, such as *Enterobacter*, *Klebsiella*, and *Citrobacter*, as well as *E. coli*. The *E. coli*, being a subset of fecal coliforms, specifically indicates more recent fecal contamination in drinking water.

The pooled prevalence of fecal contamination in POU drinking water [65.02%; 95% CI: 56.33, 73.72] in this review was lower than that reported in studies in Bangladesh (*p* = 83.78%) [37]. This discrepancy may be due to differences in HH water management practices among populations. For instance, many HHs (91.58%) in Bangladesh [37] did not treat their water, while 36.07% HHs [38], and 21% HHs [39] in Ethiopia used water treatment methods. Data collected over different years might also affect results, along with varying economic factors and access to water treatment options. For instance, POU drinking water treatment was rarely used in Ethiopia as reported in EDHS 3.0% in 2005, 8.2% in 2011, and 6.5% in 2016 [8].

On the other hand, the prevalence of fecal contamination in POU drinking water (*p* = 65.02%) in this review is in agreement with the prevalence reported in studies in Bangladesh (*p* = 62%) [40], (*p* = 70.8%) in data 38 countries [23]. This agreement likely arises from similarities in socioeconomic conditions in areas facing poor water and sanitation infrastructure. Like the current review, many of the countries examined in the latter studies [23, 40] were from Sub‐Saharan Africa (SSA) and other low and middle‐income countries (LMIC), where fecal contamination is widespread and a significant cause of waterborne diseases. The consistent results across various regions indicated that the problem is a global challenge, impacting efforts to achieve SDG 6, which aims to ensure access to clean water and sanitation for everyone.

However, the prevalence found in this review is lower than that reported in individual studies from Bangladesh (*p* = 73.96%) [41] and Nepal (*p* = 81%) [42]. This difference may be due to those studies being conducted at a single point in time, leading to higher prevalence rates, as some studies in this review reported rates that exceeded the pooled estimate.

This meta‐analysis revealed that POU drinking water in HHs with unimproved sanitation facilities had higher microbial contamination than in HHs with improved sanitation facilities [26, 29, 31]. This finding is in agreement with studies in Ghana [43] and Tanzania [44], where unimproved sanintation facilities were reported to have an increased risk of fecal contaminations in HH drinking water. This might be due to the fact that unimproved sanitation facilities promots open defication creating the full guarantee of environmental fecal contaminations. Open field defication is almost the inherent culture of most Ethiopian communities enven in HHs with imporoved sanitation facilities [45]. Open defication in both human and amimals exacerbate POU drinking water fecal contamination as this conditions facilitate water sources pollutions [46].

This review was also demonstrated that POU drinking water collected from unimproved sources [15, 27] were more likely to be contaminated with fecal bacteria than water collected from improved sources. The volume of evidences in LMICs [47], in multi‐national HH survey [23, 48], in Mozambique [49] reported similar findings, showing a strong association between unimproved water sources and increased fecal contamination in POU drinking water. This is due to both improved and unimproved water sources in environmentally contaminated areas are at an increased risk of fecal contamination due to the widespread occurrence of human and animal feces in nearby fields [50]. These findings underscore the critical importance of implementing water safety plans (WSPs) as established by the WHO. WSPs could provide a comprehensive framework for risk management in drinking‐water supplies, ensuring that all components of the water supply chain, from catchment to consumer, are thoroughly understood and monitored [51]. Additionally, the WHO has published guidelines and packages for conducting sanitary inspections and ensuring the quality of drinking water, particularly for smaller water supplies [52, 53]. However, HHs reliance on surface water as water source [15, 28] did not show significant association with fecal contamination of water at point of drinking. This finding is in contradiction with global intuitive knowledge, as surface water is generally considered more vulnerable to microbial contamination due to its exposure to environmental pollutants [54, 55]. The vulnerability of surface water for fecal contamination was proved by the independent studies included in this review [15, 28]. However, the result of this review may be attributed to the limited sample size and geographic scope of the two studies included in the analysis, which may not adequately capture the variability in surface water quality across different settings. Moreover, the absence of a significant association does not imply that surface water sources are free from fecal contamination.

Our review revealed that POU drinking water from HHs without water treatment practices [26, 29] was more likely to be contaminated with fecal bacteria compared to HHs that implemented water treatment at the HH level. A study conducted among emergency survivors in Indonesia revealed that use of chlorine solution in stored water was associated with the availability of clean drinking water in HHs [56]. Household water treatment is the last method of making POU drinking water safe, particularly in rural areas where access to piped water is limited. Studies indicated that drinking water from treated sources had an increased risk of fecal contamination at the POU within HHs, primarily due to unsafe handling and storage practices [42, 57]. This emphasizes that treatment of drinking water at any stage of the water supply system does not guarantee its safety unless it is properly used at the point of consumption. Alarmingly, the prevalence of HH water treatment in Ethiopia remains low, with less than 10% of HHs engaging in this practice [8]. In Sub‐Saharan countries, only 18% of HHs utilize adequate water treatment methods [58].

This review demonstrated that POU drinking water stored for extended period [27, 28, 29, 30, 32] was more likely to be contaminated with fecal matter compared to water stored for shorter durations. Drinking water stored for extended period may become contaminated from various sources including the storage vessel itself, hands, and the surrounding environment [59, 60]. Stored water in HHs often harbored higher levels of fecal coliform bacteria, especially when containers were not properly cleaned or were exposed to contaminants during use [61]. Water sources in rural areas are not treated with chlorine on a regular basis, making POU drinking water without residual disinfectants leading to a higher susceptibility to contamination over time [62, 63, 64]. What ever type of treatment POU drinking water received from its source, safe storage has pivotal role in preventing drinking water fecal contaminations in rural areas where water sources did not contian residual chlorine as they are not chlorinated on daily basis.

In this review, unsafe water withdrawing practice from storage container [29, 31] was significantly associated with higher POU drinking water fecal contamination compared to safe water withdrawing practices. Similar findings were reported in studies in Lima Peru [65], Kenya [66], Laos and Thailand [67], where stored water with wide mouthed container had an increased fecal contaminations as they might be contaminated during withdrawing water through dipping or scooping. Studies indicated that bowls used for dipping water from storage containers were also utilized for handwashing by dipping hands into the same bowl [68].

## 5 Strength and Limitations

This systematic review followed established protocols, including adherence to the PRISMA guidelines, ensuring transparency and reducing bias in the study selection process. However, the review included only 12 studies, which may limit the generalizability of the findings and the ability to draw stronger conclusions about the prevalence and factors associated with fecal contamination. Furthermore, majority of the studies included in this review were from Amhara region, which presents potential limitations, as variabilities in socioeconomic, cultural, and environmental factors across different regions may influence the outcomes of the studies. Most studies included in the review were cross‐sectional, which limits the ability to establish causal relationships between predictors and fecal contamination.

## 6 Conclusions and Recommendations

The fecal contamination in POU drinking water in Ethiopia was significantly high and this could challenge the country in achieving SDG 6. The review identified environmental related factors such as unimproved HH sanitation facilities, and unimproved drinking water sources; individual behavioral factors such as absence of HH water treatment practices, unsafe water withdrawal practices from storage container, and prolonged storage of POU drinking as significant predictors of higher fecal contamination in drinking water in HHs in Ethiopia. These findings indicated the urgent need in the multifaceted issues surrounding POU drinking water safety in low‐ and middle‐income countries, particularly Ethiopia, where access to clean water, hygiene and sanitation is limited. Therefore, targeted interventions, including behavioral education and programs promoting effective HH water treatment methods like chlorination, as well as safe storage and handling practices, are essential to ensure safe drinking water. The country should also invest in water source infrastructure and management to promote drinking water from improved water sources, as well as upgrade sanitation facilities, particularly in rural areas, to reduce open defecation practices to mitigate environmental contamination of water sources.

## Author Contributions

G.M.B. conception, design, acquisition of data or analysis and interpretation of data and wrote the manuscript. A.G.Y., A.T., T.D.T., S.S.T., A.A.G., G.A.Y., R.M.A., and C.H.Y. designed the study and analyzed, interpreted, and wrote the manuscript. A.G.E., A.F.A., A.M.K., H.M., Z.A.Y., A.S.E., M.A.A., B.A.M., A.S.A., and G.Y. reviewed and wrote the manuscript.

## Consent

The authors have nothing to report.

## Conflicts of Interest

The authors declare no conflicts of interest.

## Transparency Statement

The lead author Gashaw Melkie Bayeh affirms that this manuscript is an honest, accurate, and transparent account of the study being reported; that no important aspects of the study have been omitted; and that any discrepancies from the study as planned (and, if relevant, registered) have been explained.

## Supporting information


**Supporting file 1:** Key words and phrases used in different Databases for search strategy.

**Supporting file 2:** Data extraction tool to extract data from included studies.

## Data Availability

The data supporting the findings of this review are available in the Supporting Information S1: Files S1 and S2 provided with this article.

## References

[1] U. Nations , Human Rights to Water and Sanitation, 2010, https://www.unwater.org/water-facts/human-rights-water-and-sanitation.

[2] U. Nations , Resolution Adopted by the General Assembly on 28 July 2010, https://digitallibrary.un.org/record/687002?v=pdf#files.

[3] U. H. R. Office , The Right to Water; Fact Sheet 35, 2010, https://www.ohchr.org/sites/default/files/2021-09/FactSheet35en.pdf.

[4] U. N. Ethiopia , Our Work on the Sustainable Development Goals in Ethiopia, 2024, https://ethiopia.un.org/en/sdgs.

[5] F. M. Aragaw , M. W. Merid , T. M. Tebeje , M. G. Erkihun , and A. H. Tesfaye , “Unimproved Source of Drinking Water and Its Associated Factors: A Spatial and Multilevel Analysis of Ethiopian Demographic and Health Survey,” BMC Public Health 23, no. 1 (2023): 1455. 37525187 10.1186/s12889-023-16354-8 PMC10388450

[6] Ca Icf , Mini Demographic and Health Survey 2019, 2019, https://www.unicef.org/ethiopia/media/1721/file/The%202019%20Ethiopia%20Mini%20Demographic%20and%20Health%20Survey%20.pdf.

[7] Ca Icf , Ethiopia Demographic and Health Survey 2016 [FR328], 2016, https://www.dhsprogram.com/pubs/pdf/FR328/FR328.pdf.

[8] A. Geremew , B. Mengistie , J. Mellor , D. S. Lantagne , E. Alemayehu , and G. Sahilu , “Appropriate Household Water Treatment Methods in Ethiopia: Household Use and Associated Factors Based on 2005, 2011, and 2016 EDHS Data,” Environmental Health and Preventive Medicine 23 (2018): 46. 30261840 10.1186/s12199-018-0737-9 PMC6161466

[9] L. Chen , J. Jiao , S. Liu , L. Liu , and P. Liu , “Mapping the Global, Regional, and National Burden of Diarrheal Diseases Attributable to Unsafe Water,” Frontiers in Public Health 11 (2023): 1302748. 38125838 10.3389/fpubh.2023.1302748 PMC10731288

[10] J. Wolf , R. B. Johnston , A. Ambelu , et al., “Burden of Disease Attributable to Unsafe Drinking Water, Sanitation, and Hygiene in Domestic Settings: A Global Analysis for Selected Adverse Health Outcomes,” Lancet 401, no. 10393 (2023): 2060–2071. 37290458 10.1016/S0140-6736(23)00458-0 PMC10290941

[11] A. Misganaw , Y. A. Melaku , G. A. Tessema , et al., “National Disability‐Adjusted Life Years (DALYs) for 257 Diseases and Injuries in Ethiopia, 1990–2015: Findings From the Global Burden of Disease Study 2015,” Population Health Metrics 15, no. 1 (2017): 28. 28732542 10.1186/s12963-017-0146-0 PMC5521136

[12] N. E. Soboksa , S. R. Gari , A. B. Hailu , and B. M. Alemu , “Association Between Microbial Water Quality, Sanitation and Hygiene Practices and Childhood Diarrhea in Kersa and Omo Nada Districts of Jimma Zone, Ethiopia,” PLoS One 15, no. 2 (2020): e0229303. 32074128 10.1371/journal.pone.0229303 PMC7029864

[13] A. Atumo Ante , G. A. Bogale , and B. M. Adem , “Bacteriological and Physicochemical Quality of Drinking Water and Associated Risk Factors in Ethiopia,” Cogent Food & Agriculture 9, no. 1 (2023): 2219473.

[14] M. A. Usman , N. Gerber , and E. H. Pangaribowo , “Drivers of Microbiological Quality of Household Drinking Water–A Case Study in Rural Ethiopia,” Journal of Water and Health 16, no. 2 (2018): 275–288. 29676763 10.2166/wh.2017.069

[15] M. Fentie , E. Assefa , T. Tena , D. Aklog , A. Tadesse , and E. Janka , “Determinant Factors of Microbial Drinking Water Quality at the Point of Use in Rural Ethiopia: A Case Study of the South Gondar Zone,” Water 16, no. 22 (2024): 3282.

[16] B. W. Gebreegziabher , W. Y. Mergo , and B. G. Arega , “Assessment of Pathogenic and Nonpathogenic Contaminants and Their Relative Risks: The Case of Dilla Town Water Sources, Ethiopia,” Water Practice & Technology 18, no. 5 (2023): 1196–1208.

[17] T. Kimball , Chapter 3 Livestock Production Systems and Their Environmental Implications in Ethiopia (Waterville, Maine: Colby College Environmental Studies Program, 2012).

[18] O. Jones , Monitoring Sanitation and Hygiene in Rural Ethiopia: A Diagnostic Analysis of Systems, Tools and Capacity (Africa: The World Bank—Water and Sanitation Program, 2015).

[19] L. Zhang , C. Zhao , S. Cao , and B. Ye , “Effect of Water Treatment Processes on Microbial Contamination in Drinking Water in Rural Areas of the Urban Periphery,” Journal of Water, Sanitation and Hygiene for Development 14, no. 8 (2024): 734–743.

[20] D. Chalchisa , M. Megersa , and A. Beyene , “Assessment of the Quality of Drinking Water in Storage Tanks and Its Implication on the Safety of Urban Water Supply in Developing Countries,” Environmental Systems Research 6, no. 1 (2017): 12.

[21] R. A. Kristanti , T. Hadibarata , M. Syafrudin , M. Yılmaz , and S. Abdullah , “Microbiological Contaminants in Drinking Water: Current Status and Challenges,” Water, Air, & Soil Pollution 233, no. 8 (2022): 299.

[22] D. Chalchisa , M. Megersa , and A. Beyene , “Assessment of the Quality of Drinking Water in Storage Tanks and Its Implication on the Safety of Urban Water Supply in Developing Countries,” Environmental Systems Research 6 (2018): 12.

[23] T. M. Santos , A. Wendt , C. V. N. Coll , M. A. Bohren , and A. J. D. Barros , “ *E. coli* Contamination of Drinking Water Sources in Rural and Urban Settings: An Analysis of 38 Nationally Representative Household Surveys (2014–2021),” Journal of Water and Health 21, no. 12 (2023): 1834–1846. 38153715 10.2166/wh.2023.174

[24] M. J. Page , L. Shamseer , and A. C. Tricco , “Registration of Systematic Reviews in PROSPERO: 30,000 Records and Counting,” Systematic Reviews 7, no. 1 (2018): 32. 29463298 10.1186/s13643-018-0699-4 PMC5819709

[25] R. Bain , R. Cronk , J. Wright , H. Yang , T. Slaymaker , and J. Bartram , “Fecal Contamination of Drinking‐Water in Low‐And Middle‐Income Countries: A Systematic Review and Meta‐Analysis,” PLoS Medicine 11, no. 5 (2014): e1001644. 24800926 10.1371/journal.pmed.1001644 PMC4011876

[26] Y. Alemeshet Asefa , B. M. Alemu , N. Baraki , D. Mekbib , and D. A. Mengistu , “Bacteriological Quality of Drinking Water From Source and Point of Use and Associated Factors Among Households in Eastern Ethiopia,” PLoS One 16, no. 10 (2021): e0258806. 34653216 10.1371/journal.pone.0258806 PMC8519474

[27] A. Getachew , A. Tadie , D. H. Chercos , et al., “Bacteriological Quality of Household Drinking Water in North Gondar Zone, Ethiopia; a Community‐Based Cross‐Sectional Study,” Applied Water Science 11, no. 12 (2021): 189.

[28] G. K. Mekonnen , B. Mengistie , G. Sahilu , W. Mulat , and H. Kloos , “Determinants of Microbiological Quality of Drinking Water in Refugee Camps and Host Communities in Gambella Region, Ethiopia,” Journal of Water, Sanitation and Hygiene for Development 9, no. 4 (2019): 671–682.

[29] G. Berihun , M. Abebe , S. Hassen , et al., “Drinking Water Contamination Potential and Associated Factors Among Households With Under‐Five Children in Rural Areas of Dessie Zuria District, Northeast Ethiopia,” Frontiers in Public Health 11 (2023): 1199314. 37361152 10.3389/fpubh.2023.1199314 PMC10289289

[30] A. Getachew , A. Tadie , D. H. Chercos , and T. Guadu , “Level of Faecal Coliform Contamination of Drinking Water Sources and Its Associated Risk Factors in Rural Settings of North Gondar Zone, Ethiopia: A Cross‐Sectional Community Based Study,” Ethiopian Journal of Health Sciences 28, no. 2 (2018): 227–234. 29983520 10.4314/ejhs.v28i2.14 PMC6016343

[31] T. A. Birhan , B. Destaw , and H. Dagne , Quality of household Drinking Water and Its associated Risk Factors in Flood‐Prone settings of Northwest Ethiopia: A cross‐sectional community‐based study. 2022. 10.1016/j.heliyon.2023.e15072 PMC10161371 37151633

[32] A. A. Aydamo , S. Robele Gari , and S. T. Mereta , “Seasonal Variations in Household Water Use, Microbiological Water Quality, and Challenges to the Provision of Adequate Drinking Water: A Case of Peri‐Urban and Informal Settlements of Hosanna Town, Southern Ethiopia,” Environmental Health Insights 18 (2024): 11786302241238940. 38525297 10.1177/11786302241238940 PMC10958793

[33] A. Keleb , A. Ademas , T. Sisay , M. Lingerew , and M. Adane , “Bacteriological Quality of Bottled Drinking Water and Municipal Tap Water in Northeastern Ethiopia,” Frontiers in Environmental Science 10 (2022): 828335.

[34] H. Feleke , G. Medhin , H. Kloos , J. Gangathulasi , and D. Asrat , “Household‐Stored Drinking Water Quality Among Households of Under‐Five Children With and Without Acute Diarrhea in Towns of Wegera District, in North Gondar, Northwest Ethiopia,” Environmental Monitoring and Assessment 190 (2018): 669. 30353421 10.1007/s10661-018-7033-4 PMC6208974

[35] K. Amenu , M. Spengler , A. Markemann , and A. V. Zárate , “Microbial Quality of Water in Rural Households of Ethiopia: Implications for Milk Safety and Public Health,” Journal of Health, Population, and Nutrition 32, no. 2 (2014): 190–197. 25076657 PMC4216956

[36] World Health Organizations ., *Drinking‐Water Key Facts*, *a*ccessed *through*, 2022, https://www.who.int/news-room/fact-sheets/detail/drinking-water.

[37] J. R. Khan , M. B. Hossain , P. A. Chakraborty , and S. K. Mistry , “Household Drinking Water *E. coli* Contamination and Its Associated Risk With Childhood Diarrhea in Bangladesh,” Environmental Science and Pollution Research 29, no. 21 (2022): 32180–32189. 35015232 10.1007/s11356-021-18460-9

[38] A. Tamene , A. Habte , D. Woldeyohannes , et al., “Water Treatment at the Point‐of‐Use and Treatment Preferences Among Households in Ethiopia: A Contemporaneous Systematic Review and Meta‐Analysis,” PLoS One 17, no. 10 (2022): e0276186. 36301990 10.1371/journal.pone.0276186 PMC9612552

[39] B. Desye , A. H. Tesfaye , G. Berihun , T. Sisay , C. Daba , and L. Berhanu , “Household Water Treatment Practice and Associated Factors in Ethiopia: A Systematic Review and Meta‐Analysis,” PLoS One 18, no. 6 (2023): e0285794. 37289814 10.1371/journal.pone.0285794 PMC10249828

[40] J. R. Khan and K. S. Bakar , “Spatial Risk Distribution and Determinants of *E. coli* Contamination in Household Drinking Water: A Case Study of Bangladesh,” International Journal of Environmental Health Research 30, no. 3 (2020): 268–283. 30924350 10.1080/09603123.2019.1593328

[41] Z. H. Mahmud , M. S. Islam , K. M. Imran , et al., “Occurrence of *Escherichia Coli* and Faecal Coliforms in Drinking Water at Source and Household Point‐of‐Use in Rohingya Camps, Bangladesh,” Gut Pathogens 11, no. 1 (2019): 52. 31695751 10.1186/s13099-019-0333-6 PMC6824040

[42] D. Daniel , A. Diener , J. van de Vossenberg , M. Bhatta , and S. J. Marks , “Assessing Drinking Water Quality at the Point of Collection and Within Household Storage Containers in the Hilly Rural Areas of Mid and Far‐Western Nepal,” International Journal of Environmental Research and Public Health 17, no. 7 (2020): 2172. 32218157 10.3390/ijerph17072172 PMC7178164

[43] S. T. McGarvey , J. Buszin , H. Reed , et al., “Community and Household Determinants of Water Quality in Coastal Ghana,” Journal of Water and Health 6, no. 3 (2008): 339–349. 19108554 10.2166/wh.2008.057 PMC3696883

[44] M. C. Mattioli , A. B. Boehm , J. Davis , A. R. Harris , M. Mrisho , and A. J. Pickering , “Enteric Pathogens in Stored Drinking Water and on Caregiver's Hands in Tanzanian Households With and Without Reported Cases of Child Diarrhea,” PLoS One 9, no. 1 (2014): e84939. 24392161 10.1371/journal.pone.0084939 PMC3879350

[45] A. Temesgen , M. Molla Adane , A. Birara , and T. Shibabaw , “Having a Latrine Facility Is Not a Guarantee for Eliminating Open Defecation Owing to Socio‐Demographic and Environmental Factors: The Case of Machakel District in Ethiopia,” PLoS One 16, no. 9 (2021): e0257813. 34591873 10.1371/journal.pone.0257813 PMC8483416

[46] O. T. W. Mochware , M. L. Thaoge‐Zwane , and M. N. B. Momba , “Applying Microbial Source Tracking Techniques for Identification of Pathways of Faecal Pollution From Water Sources to Point of Use in Vhembe District, South Africa,” Water 16, no. 14 (2024): 2014.

[47] R. Bain , R. Cronk , J. Wright , H. Yang , T. Slaymaker , and J. Bartram , “Fecal Contamination of Drinking‐Water in Low‐ and Middle‐Income Countries: A Systematic Review and Meta‐Analysis,” PLoS Medicine 11, no. 5 (2014): e1001644. 24800926 10.1371/journal.pmed.1001644 PMC4011876

[48] R. Bain , R. Johnston , S. Khan , A. Hancioglu , and T. Slaymaker , “Monitoring Drinking Water Quality in Nationally Representative Household Surveys in Low‐and Middle‐Income Countries: Cross‐Sectional Analysis of 27 Multiple Indicator Cluster Surveys 2014‐2020,” Environmental Health Perspectives 129, no. 9 (2021): 097010. 34546076 10.1289/EHP8459 PMC8454503

[49] A. R. Yang , J. M. Bowling , C. E. Morgan , J. Bartram , and G. L. Kayser , “Predictors of Household Drinking Water *E. coli* Contamination: Population‐Based Results From Rural Areas of Ghana, Malawi, Mozambique, Niger, Rwanda, Uganda, and Zambia,” International Journal of Hygiene and Environmental Health 264 (2025): 114507. 39662127 10.1016/j.ijheh.2024.114507 PMC12834120

[50] A. Shaheed , J. Orgill , M. A. Montgomery , M. A. Jeuland , and J. Brown , “Why? Improved? Water Sources Are Not Always Safe,” Bulletin of the World Health Organization 92 (2014): 283–289. 24700996 10.2471/BLT.13.119594 PMC3967570

[51] World Health Organizations . Water Safety Plan Manual: Step‐by‐Step Risk Management for Drinking‐Water Suppliers, Second Edition, 2023, https://iris.who.int/handle/10665/366148.

[52] World Health Organizations . Guidelines for Drinking‐Water Quality: Small Water Supplies, 2023, https://iris.who.int/handle/10665/375822.

[53] World Health Organizations . Sanitary Inspection Packages: A Supporting Tool for the “Guidelines for Drinking‐Water Quality: Small Water Supplies”, 2023, https://iris.who.int/handle/10665/375824.

[54] A. Taiwo , O. O. Olujimi , O. Bamgbose , and T. A. Arowolo , “Surface Water Quality Monitoring in Nigeria: Situational Analysis and Future Management Strategy,” Water Quality Monitoring and Assessment 13 (2012): 301–320.

[55] D. L. Weller , C. M. Murphy , S. Johnson , et al., “Land Use, Weather, and Water Quality Factors Associated With Fecal Contamination of Northeastern Streams That Span an Urban‐Rural Gradient,” Frontiers in Water 3 (2022): 741676.

[56] S. K. Gupta , A. Suantio , A. Gray , et al., “Factors Associated With *E. coli* Contamination of Household Drinking Water Among Tsunami and Earthquake Survivors, Indonesia,” American Journal of Tropical Medicine and Hygiene 76, no. 6 (2007): 1158–1162. 17556629

[57] G. Joseph , S. Haque , N. Moqueet , and Y. R. Hoo , 2019. Children Need Clean Water to Grow: E. Coli Contamination of Drinking Water and Childhood Nutrition in Bangladesh. Coli Contamination of Drinking Water and Childhood Nutrition in Bangladesh (November 7, 2019). World Bank Policy Research Working Paper, 9054.

[58] A. Geremew and Y. T. Damtew , “Household Water Treatment Using Adequate Methods in Sub‐Saharan Countries: Evidence From 2013–2016 Demographic and Health Surveys,” Journal of Water, Sanitation and Hygiene for Development 10, no. 1 (2020): 66–75.

[59] I. Slavik , K. R. Oliveira , P. B. Cheung , and W. Uhl , “Water Quality Aspects Related to Domestic Drinking Water Storage Tanks and Consideration in Current Standards and Guidelines Throughout the World – A Review,” Journal of Water and Health 18, no. 4 (2020): 439–463. 32833673 10.2166/wh.2020.052

[60] T. Navab‐Daneshmand , M. N. D. Friedrich , M. Gächter , et al., “ *Escherichia coli* Contamination Across Multiple Environmental Compartments (Soil, Hands, Drinking Water, and Handwashing Water) in Urban Harare: Correlations and Risk Factors,” American Journal of Tropical Medicine and Hygiene 98, no. 3 (2018): 803–813. 29363444 10.4269/ajtmh.17-0521 PMC5930891

[61] M. Manga , T. G. Ngobi , L. Okeny , et al., “The Effect of Household Storage Tanks/Vessels and User Practices on the Quality of Water: A Systematic Review of Literature,” Environmental Systems Research 10, no. 1 (2021): 18.

[62] A. Admasie , K. Abera , and F. W. Feleke , “Household Water Treatment Practice and Associated Factors in Rural Households of Sodo Zuria District, Southern Ethiopia: Community‐Based Cross‐Sectional Study,” Environmental Health Insights 16 (2022): 11786302221095036. 35479294 10.1177/11786302221095036 PMC9036349

[63] I. Evance , F. Ongeta , S. Chebet , et al., “Assessing the Factors Affecting the Use of Chlorine Dispensers for Household Water Treatment in Rural Kenya, Malawi, and Uganda,” Journal of Water, Sanitation and Hygiene for Development 15 (2024): 84–94.

[64] R. A. Li , J. A. McDonald , A. Sathasivan , and S. J. Khan , “Disinfectant Residual Stability Leading to Disinfectant Decay and By‐Product Formation in Drinking Water Distribution Systems: A Systematic Review,” Water Research 153 (2019): 335–348. 30743084 10.1016/j.watres.2019.01.020

[65] W. E. Oswald , G. Lescano , C. Bern , M. M. Calderon , L. Cabrera , and R. H. Gilman , “Fecal Contamination of Drinking Water Within Peri‐Urban Households, Lima, Peru,” American Journal of Tropical Medicine and Hygiene 77, no. 4 (2007): 699–704. 17978074

[66] P. Kirianki , J. Othira , and S. Kiruki , Analysis of Microbial Quality of Drinking Water in Njoro Sub‐County, Kenya. 2019.

[67] N. Vannavong , H. J. Overgaard , T. Chareonviriyaphap , et al., “Assessing Factors of *E. coli* Contamination of Household Drinking Water in Suburban and Rural Laos and Thailand,” Water Supply 18, no. 3 (2017): 886–900.

[68] J. Eshcol , P. Mahapatra , and S. Keshapagu , “Is Fecal Contamination of Drinking Water After Collection Associated With Household Water Handling and Hygiene Practices? A Study of Urban Slum Households in Hyderabad, India,” Journal of Water and Health 7, no. 1 (2009): 145–154. 18957783 10.2166/wh.2009.094

