# Supplementary material for: Fecal Contamination in Point of Use (POU) Drinking Water and Its Associated Factors in Ethiopia: Systematic Review and Meta‐Analysis; “Implications for SDG 6 and WASH Interventions”
Source: Health Sci Rep. 2025 Nov 23;8(11):e71445. doi: 10.1002/hsr2.71445 (PMC12641107; doi:10.1002/hsr2.71445)
Supplement: Supplementary file 1 — Supporting file 1: Key words and phrases used in different Databases for search strategy. [file HSR2-8-e71445-s001.docx]

Supplementary file 1: Key words and phrases used in different Databases for search strategy

|  | Databases | No of articles |
| --- | --- | --- |
|  | PubMed |  |
| #1 | (Drinking water) OR (Water) | 1,369,082 |
| #2 | (Microbial quality) OR (Bacteriological quality) OR (Fecal contamination) OR (E.coli contamination) | 60,430 |
| #3 | (Point of use) OR (point of consumption) OR (point of Drinking) | 1,375,096 |
| #4 | (Associated factors)) OR (Risk Factors)) OR (Determinant Factors)) | 4,778,456 |
| #5 | (Ethiopia) | 47,628 |
|  | #1 & #2 & #3 & #4 & #5 | 1000 |
|  | Google Scholar |  |
| #1 | "Drinking water" OR “Water" | 748,000 |
| #2 | "Microbial quality" OR "Bacteriological quality" OR "Fecal contamination" OR "E coli contamination" | 26,400 |
| #3 | (Point of use) OR (point of consumption) OR (point of Drinking) | 19,000 |
| #4 | "Associated factors" OR "Risk Factors" OR "Determinant Factors" | 17,800 |
| #5 | "Ethiopia" | 1,120,000 |
|  | #1 & #2& #3& #4 & #5 | 558 |
|  | African Journal Online |  |
| #1 | (Drinking water) OR (Water) | 78,802 |
| #2 | (Microbial quality) OR (Bacteriological quality) OR (Fecal contamination) OR (E.coli contamination) | 95,823 |
| #3 | (Point of use) OR (point of consumption) OR (point of Drinking) | 162,836 |
| #4 | (Associated factors)) OR (Risk Factors)) OR (Determinant Factors)) | 153,848 |
| #5 | (Ethiopia) | 25,960 |
|  | #1 & #2& #3& #4 & #5 | 3,734 |
|  | Web of science |  |
| #1 | (Drinking water) OR (Water) | 4,607,558 |
| #2 | (Microbial quality) OR (Bacteriological quality) OR (Fecal contamination) OR (E.coli contamination) | 178,560 |
| #3 | (Point of use) OR (point of consumption) OR (point of Drinking) | 0818,426 |
| #4 | (Associated factors)) OR (Risk Factors)) OR (Determinant Factors)) | 4,287,840 |
| #5 | (Ethiopia) | 89,886 |
|  | #1 & #2& #3& #4 & #5 | 10 |
|  | Cochrane library |  |
| #1 | (Drinking water) OR (Water) | 44014 |
| #2 | (Microbial quality) OR (Bacteriological quality) OR (Fecal contamination) OR (E.coli contamination) | 1702 |
| #3 | (Point of use) OR (point of consumption) OR (point of Drinking) | 66702 |
| #4 | (Associated factors)) OR (Risk Factors)) OR (Determinant Factors)) | 140,616 |
| #5 | (Ethiopia) | 1692 |
|  | #1 & #2& #3& #4 & #5 | 13 |
|  | Scopus |  |
| #1 | (Drinking water) OR (Water) | 174,889 |
| #2 | (Microbial quality) OR (Bacteriological quality) OR (Fecal contamination) OR (E.coli contamination) | 187 |
| #3 | (Point of use) OR (point of consumption) OR (point of Drinking) | 9,283 |
| #4 | (Associated factors)) OR (Risk Factors)) OR (Determinant Factors)) | 1,018,966 |
| #5 | (Ethiopia) | 122,338 |
|  | #1 & #2& #3& #4 & #5 | 1 |
